# Patient involvement in clinical trials: motivation and expectations differ between patients and researchers involved in a trial on urinary tract infections

**DOI:** 10.1186/s40900-019-0145-3

**Published:** 2019-04-01

**Authors:** Imke Schilling, Heike Behrens, Claudia Hugenschmidt, Jennifer Liedtke, Guido Schmiemann, Ansgar Gerhardus

**Affiliations:** 1 Department for Health Services Research, Institute of Public Health and Nursing Research, University of Bremen, Grazer Straße 4, 28359 Bremen, Germany; 2 Health Sciences Bremen, University of Bremen, 28359 Bremen, Germany

**Keywords:** Clinical trial, Patient and public involvement, PPI, Patient engagement, Patient board, Motivation, Motives, Expectations, Qualitative research

## Abstract

**Plain English summary:**

Patients should be involved in the design, conduct and dissemination of research that affects them. Patient involvement leads to empowerment and enhances the quality of research. Differing motives and expectations between researchers and patients involved can hamper involvement. We wanted to learn more about patients’ and researchers’ motives and expectations in order to improve the benefits of involvement for all parties. We implemented a patient board with ten patients and five researchers for a trial on urinary tract infections (UTIs). We asked each patient and researcher about his or her motivation and expectations regarding the patient board. We found that patients’ motivations included the wish to improve the treatment of UTIs, to support patient involvement as a principle, and to enhance the benefit of others. Furthermore they were interested in learning how a patients’ board works and in exchanging with peers and scientists. In addition, a (modest) monetary incentive for involvement was welcomed.Researchers were motivated by the possibility to improve research and to contribute to the empowerment of patients. They also wanted to enhance their career opportunities, to learn more about patient involvement and to meet the increasing demand for it. Some patients expressed insecurity about their roles and tasks in the patient board. Among the researchers, some envisaged a rather passive role for themselves in the patient board while others expected to take over a more active role. Researchers emphasized that the ways and the means of communication between the researchers and the patients should be explicitly discussed.

**Abstract:**

**Background**

It has been increasingly recognized that patients should be actively involved in the design, conduct and dissemination of research. Besides empowering patients and democratizing research, involvement can enhance the quality of research and the development of equitable healthcare solutions. Differing motives and expectations between researchers and involved patients can hamper the conduct of involvement. However, little is known about patients’ and researchers’ motivations for involvement. Our aim was to study the motivation and expectations of patients and researchers towards patient and public involvement (PPI).

**Methods**

We implemented a patient board comprising ten patients and five researchers for a randomized controlled trial on the treatment of urinary tract infections (UTI). Prior to the first board meeting, we conducted telephone interviews with all researchers and patients regarding their motivation for involvement in the patient board and their expectations. The interviews were analyzed using thematic qualitative text analysis.

**Results**

Patients’ motivations included interest in improving UTI treatment, in supporting PPI, engaging for the benefit of others, exchanging with peers and scientists as well as in the methods of the board and the monetary incentive. Researchers wanted to improve research, enhance their professional development, empower patients, meet the formal demand for PPI, and learn about PPI. Regarding expectations, patients expressed insecurities about their roles, tasks and topics of discussion. They wished for an open exchange and hoped their involvement would make an impact. Researchers’ expectations for their own roles ranged between being a rather passive supporting force and active engagement in the board. The question of how to ensure the communication between the trial team and the patient board was of high importance for the researchers.

**Conclusions**

Patients’ and researchers’ motives and expectations were similar in some aspects but differed regarding agenda setting and understanding of their roles. Getting to know patients’ and researchers’ motivations and expectations at the beginning allowed us to anticipate potential conflicts or disappointments early on and to take them into consideration during the conduct of our PPI.

**Electronic supplementary material:**

The online version of this article (10.1186/s40900-019-0145-3) contains supplementary material, which is available to authorized users.

## Background

In recent years, patients, researchers and research funding organizations have increasingly recognized that patients should be actively involved in the design, conduct and dissemination of clinical research [1–4]. Normative and substantive rationales [5, 6] argue for “research being carried out ‘with’ or ‘by’ members of the public rather than ‘to’, ‘about’ or ‘for’ them” [7]. Normatively, patient and public involvement (PPI) is justified as an end in itself. It supports the empowerment of patients and leads to a democratization of research processes [4, 8, 9]. Substantive arguments focus on the consequences of PPI and see it as a means to enhance the quality and relevance of research [10]. Also, studies have shown discrepancies between patients’ and researchers’ preferences, with the two groups ranking outcomes differently [11–13]*.* PPI is argued to enhance the focus of clinical trials on the needs of patients, improve recruitment, raise the quality of findings and help their dissemination [4, 8, 9, 14].

While the frequency with which PPI is being demanded and conducted is increasing [15], different papers report that even when people are willing to get involved in PPI, the effective realization of involvement can be hampered by differing ideas of PPI or the misinterpretation of aims and expectations among and between the parties involved [5, 16]. The values and ideas patients and researchers hold with regard to PPI influence the approaches used to conduct the involvement and the impact that results from it [5]. A mismatch can inhibit successful involvement [5]. To date information on the motivations and expectations of patients and researchers when getting involved in PPI is mostly anecdotally described in studies reporting on PPI [4, 14, 17, 18]. There have been few studies, which explicitly investigated motives and expectations for PPI: Tarpey reviewed articles on PPI and found personal and social reasons that influence the motivation of patients or the public to get involved in research [19]. Personal reasons refer to having a voice as well as the opportunity for personal development in terms of confidence, skills and self-esteem [19]. Social aspects reflected the wish to change research and services for the benefit of others [19]. Thompson et al. confirmed these results in a trial with patients and caregivers that were involved in cancer research [20]. Boaz et al. asked researchers about their attitude towards PPI [21]. They found that researchers’ attitudes ranged from the positive expectation that PPI improves research, to PPI being something that needs to be done to comply with a formal demand. While we found studies that described patients’ or researchers’ ideas of why to conduct PPI, we did not find any study that systematically investigated and contrasted both patients’ and researchers’ motives and expectations. The literature indicates that the view on PPI may vary quite strongly, from PPI being a value in itself, to PPI being a burden that needs to be carried [21–23]. To prevent conflicts, Gradinger et al. recommend that the parties involved in PPI explicitly reflect their positions and motives beforehand [5].

Our aim was to study the motivations and expectations patients and researchers in Germany have when establishing PPI. Our research questions were: (a) What motivates patients and researchers to get involved in a patient board for a clinical trial? (b) Which expectations do they associate with their involvement?

In contrast to countries such as the United Kingdom, which has the expert group INVOLVE, or the United States with the Patient Centered Outcome Research Institute (PCORI), a structure that supports patients being regularly involved in the design, conduct and dissemination of (clinical) research has yet to be established in Germany.

For consistency and after discussion with patient co-authors, we use the term ‘patient’ throughout the article when referring to the people that bring specific health related experiences into research as members of the public as it seems to be the most explicit and easily understood term. We are aware that other terms such as ‘service users’ would also fit and may better convey the active role that PPI strives for. Furthermore we used the term ‘patient and public involvement’ respectively its abbreviation PPI to describe the involvement of the ‘patients’ in the conduct of the UTI trial, even though we are aware that the women that bring own experiences with UTIs into research are only a part of those who could get involved as the ‘public’. We did not involve further perspectives as potential patients or caregivers. The term ‘researchers’ refers to all those that comprise the clinical trial team, e.g. academic researchers, physicians and study nurses.

According to Heckhausen and Heckhausen [24] and Rheinberg and Vollmeyer [25], we define motivation as a product of personal and situational factors. Personal factors are needs, motives and aims that influence motivation and give reason to get into action. Situations influence motivation by offering incentives that may be connected to the action itself, its outcomes or expected consequences [24]. Situations may incite people to get into action if the expectations align with personal preferences and aims [25]. The incentive decreases if a person expects the positive outcomes or consequences to occur without his or her own contribution [24].

## Methods

### Establishing a patient board

We aimed at studying the motivations and expectations patients and researchers in Germany have when establishing PPI. This study was conducted within the context of a randomized controlled trial (RCT) comparing herbal treatment to antibiotic treatment for uncomplicated urinary tract infections (UTI) in women (EudraCT No. 2016–000477-21) [26]. During the proposal phase of the clinical RCT, the researchers involved assessed patient perspectives using the literature and conducting a one-off discussion with patients’ representatives. To establish a long-term involvement of patients, they invited the authors of this article to establish a patient board that would support the conduct of the clinical RCT from the preparation phase until the dissemination of results. The patient board comprised ten patients and five researchers. The ten patients met regularly (2–3 times a year) to exchange views and discuss relevant aspects of the trial. One researcher also participated in all board meetings, while the other four researchers were invited to join the board meetings as they wished. The mode of cooperation within the patient board was discussed and agreed upon during the first patient board meeting. For every patient board meeting they participated in, the patients received an allowance of 50 Euros. Researchers did not receive any allowances.

### Selection of patient board members

All patients that got involved in the board had to be female and had to have suffered from UTI. They were deliberately selected to possess diverse characteristics with regard to age, educational background and experiences with clinical research [14, 27–29]. Patients were contacted for involvement via general medical practices (*N* = 4), an online advertisement (*N* = 5) and mutual acquaintances (*N* = 1). Those who showed interest received information on the following aspects of the project in writing: the patient board and that it was a qualitative study. This served to ensure fully informed consent. Additionally, the patients gave written consent. The participating members of the trial team (‘researchers’) (N = 5) were asked for participation based on their involvement in the UTI trial by IS. They also received written information on the project and gave written consent. Both patients and researchers filled out short questionnaires in which socio-demographic data and information on their experiences with UTIs (patients) and PPI (patients and researchers) were collected.

### Telephone interviews with patient board members

A qualitative research design was used to capture the members’ motivations for involvement in the patient board. Based on the theory that motivation results from the interaction of personal and situational factors [24, 25] and the findings on motivational aspects occasionally reported in articles on PPI, we developed two semi-structured interview guidelines: one for the patients and one for the researchers. The interview guidelines were discussed and agreed upon among IS, JL, GS and AG and were pretested with three women who were not part of the patient board. The interviews started with an open question on why the patients had agreed to get involved in the patient board. Further exploratory and ad-hoc questions reflected on personal and situational factors, aims for their involvement, thematic interests, expectations regarding how they would work on and contribute to the board, and experiences with patient involvement and clinical trials.

We conducted telephone interviews with each member of the patient board for practical reasons. Three out of ten patients lived out of town and four out of five researchers lived far away. Therefore telephone interviews were a more feasible method than face-to-face interviews. The semi-structured telephone interviews were conducted by the first author of this manuscript (IS), who is female, according to Witzel’s method for problem-centered interviews [30]. To help them get acquainted with their role, patients were given background material on clinical research, the specific UTI trial and on patient involvement, prior to the interviews. The interviews took place after the patients and researchers had given informed consent but before the first meeting of the patient board. At the end of each interview, the interviewer paraphrased and summarized the main findings of the interview and asked for direct oral feedback on her interpretation. Thus, the interviewees were able to add further information and/or make corrections.

Each interview was audio-recorded and first impressions noted in a postscript. The interviews with researchers lasted 15 to 29 min (19:54 min mean) and with patients nine to 15 min (12:14 min mean). In addition to the allowances for patient board meetings, patients also received an expense allowance of 50 Euros for the telephone interviews; researchers did not receive allowances.

### Analysis

The interviews were transcribed and pseudonymized by two student assistants and checked for accuracy by IS. They were not returned to the involved patients and researchers for further commenting. The transcripts were imported into MAXQDA (Version 11, VERBI GmbH, Berlin, Germany) for analysis. Data analysis was conducted by IS and JL following Kuckartz’s seven steps of thematic qualitative text analysis [31]. Based on textual work with each case and our interview guidelines (step 1), primary categories for both patients and researchers were developed (step 2) and all transcripts coded with these categories (step 3). Using the code memos and all text passages of each category (step 4), the preliminary categories were elaborated into final categories and subcategories (step 5) and the whole material coded again (step 6). The analysis for each category was conducted separately for patients and researchers (step 7). Interpersonal perception was reviewed through discussion within the author team. The findings were used to structure our results section. As the interview transcripts were in German, citations used in this article were translated into English by IS.

Ethical approval was obtained from the ethics committee of the University of Bremen (Germany). The author team conducted the study. IS, JL, GS and AG are academic researchers with experience in conducting qualitative research. HB and CH are members of the said patient board. We followed the consolidated criteria for reporting qualitative research (COREQ) (see Additional file 1) [32]. Furthermore, we followed the GRIPP2 checklist for the reporting of PPI (see Additional file 2) [33].

### Involving patients’ perspectives in the analysis and discussion of results

To involve the patients’ perspective in the analysis, we discussed the results of the telephone interviews in the first patient board meeting with the six patients and one researcher who were present. As only six of the ten patients were present at the meeting, we also sent an overview of the results to all patients (and researchers) and asked for further comments. The results were confirmed during the discussion in the first patient board with the patients and the researcher, and there were no suggestions for changes. In addition, we asked all ten patients if some of them were interested in co-authoring this article. Two patients (HB and CH) expressed deeper interest to involve their perspectives in the preparation of the article to help with learning more about PPI. With them we intensively discussed the draft of the article. The patient co-authors contributed by giving valuable insights to the discussion of the results.

## Results

All 15 members of the patient board (patients *N* = 10, researchers *N* = 5) participated in the telephone interviews. Table 1 presents the characteristics of the involved patients and researchers.

**Table 1:** Characteristics of involved patients and researchers

Patients (*N* = 10)			Researchers (*N* = 5)		
Age	20–34	3	Age	20–34	1
	35–49	2		35–49	3
	50–64	5		50–64	1
Educational level	Higher education	6	Gender	Female	3
	Secondary ed.	4		Male	2
Employment status	Employed	6	Number of previous clinical trials conducted	0	1
	Student	1		2	4
	Retired	1		2	4
	Not employed	2	Task in clinical trial	Coordination	4
UTI within last 12 months	0x	1		Study nurse	1
	1-2x	7			
	3-5x	1			
	> 5x	1			

### Patients’ and researchers’ background

All women who got involved as ‘patients’ felt restricted by UTIs in their everyday life. They described their UTI experiences as very burdensome and a “fight”, adding that they felt “defenseless” (P1:31). The patients reported that they had learned a lot about the prevention and treatment of UTIs through their own experiences and research. One patient had previous experience as a participant in a UTI trial. Four of the five researchers were trained physicians and the fifth was a trained nurse. All but one of the researchers had previous experience with clinical trials. Neither the patients nor the researchers had previous experience with systematic PPI.

## Motivation

### Reasons for involvement and aims

Both patients’ and researchers’ motivation for involvement arose from a mix of different reasons. Table 2 gives an overview of the reasons patients and researchers named. While some reasons were to be expected as they align with the “classic” argumentation for PPI (empowerment of patients, democratization of research processes, enhancement of the quality and relevance of research), others were not expected but are equally plausible.

**Table 2:** Reasons for involvement

	Patients	Researchers
“Classic” PPI arguments normative and substantive	• Improve treatment • Support PPI	• Improve research • Empower patients
Further arguments	• Engage for the benefit of others • Exchange with peers and scientists • Interest in methods of the board • Monetary incentive	• Professional development • Meet formal demand for PPI • Learn about PPI

#### Patients

For the patients we categorized six reasons for getting involved. Patients were interested in 1) improving UTI treatment, 2) supporting PPI, 3) engaging for the benefit of others, 4) exchanging with peers and scientists, 5) the methods of the board and/or 6) the monetary incentive. The first two reasons align with the general argumentation for PPI.

1. Improving UTI treatment

Patients wanted to enhance further development in UTI treatment by contributing their own perspectives towards research. They generally aimed for the development of new drugs as alternatives to antibiotics as well as individuality in treatment. They got involved hoping that “as a person concerned with UTI, one could perhaps contribute to a medicine or something that would help the women, being refined” (P10:2). Patients equated researchers with physicians and saw differences between their own perspectives and those of physicians. While they assigned the physicians scientific and medical knowledge they “[…] expect[ed] holism from the patient board” (P4:2).

2) Supporting PPI

A more general reason for patients to get involved was to support the approach of empowering patients and involving their perspectives into clinical research processes. Patients did not know PPI in advance but thought it was a good approach. One of them stated “I think the idea, to involve the experiences of the participants and [have] another perspective than the medical perspective. I think that is a great idea” (P3:2).

3) Engaging for the benefit of others

In addition to the first two reasons expected for PPI, the patients stated other reasons that motivated them. While most patients got involved to “help myself and others by that” (P7:2), some had the explicit motivation to contribute their perspectives for the benefit of others. They mentioned that they like to be active in the community - “I am a very engaged person and, yes, I like to engage in issues where I have the impression I can have an influence” (P1:2) – or found the board while “looking for useful activities”, as someone who was “going to retire soon” (P9:2). Some engaged in other contexts, e.g. the community or a works council.

4) Exchanging with peers and scientists

Furthermore, patients were motivated by the idea of having an exchange with peers and scientific experts: “I’ve been fighting with recurring UTIs for about ten years, … and with medicine, I’m not making any progress, …so I am interested in how others feel, how they handle it” (P2:2). One woman said she was uncertain about trusting her own judgment. She wanted to use the exchange to compare her experiences with that of others and gain reassurance through that. Another one got involved to promote her self-development by learning more about herself and feeling motivated to share her opinion.

5) The methods of the board

Patients were also interested in participating in the process of the patient board and the clinical RCT so they could learn about the board’s methods. For instance, one young patient hoped to be able to use the new knowledge about the functioning of the board for her job: “It is not exactly the topic that is of burning interest for me, but more the method” (P5:2). Another woman stated: “Patient board, research, a whole new area for me, […] I find that really good and exciting as it links so many things that have determined my life for so long due to the UTIs” (P6:2).

6) The monetary incentive

The importance of the allowances (50 Euros per board meeting) differed among the patients. For some, the allowance added to their motivation for involvement: “Next to the personal concern and the interest in the topic, for me the allowances are a further reason [for involvement]” (P4:2). For others they were a welcome gratification – “I am happy for the small token” (P10:2) –, or a sign of appreciation for the time they invest and allowance for their travel expenses. The general feeling was that the allowance would have to be much higher for it to be the main reason for involvement.

#### Researchers

For the researchers we categorized five reasons that motivated them to support the patient board. In accordance with classical PPI aims they wanted to 1) improve research and/or 2) empower patients. Further motivations mentioned were to 3) enhance their professional development 4) meet the formal demand for PPI and/or 5) learn about PPI.

1. Improve research

Researchers wanted to learn about patients’ perspectives so as to be able to consider them in the research process and improve research. They felt that their perspectives differ from patients’ perspectives and did not feel able to represent patients’ views – “a perspective that you can’t have yourself, because you are on the other side of the whole scientific… or the research team” (R2:3). Furthermore, the researchers lacked personal experience with the condition in question: “The patients’ point of view is an important part of research work. If one doesn’t incorporate it, I believe the value of the research findings is not very high” (R2:3). Researchers saw PPI as an approach to enhance the quality of research. They aimed for better intelligibility of trial procedures and documents so as to improve the practicability for patients and raise acceptance. By learning about patients’ views and priorities, researchers wanted to gain more clarity and new ideas for themselves, help implement trial results and improve future research. According to the researchers, PPI adds a ‘reality check’ to research, and patients were perceived to serve as a link between practice and research (working in both directions). One researcher went on to describe the benefit of involvement more generally as profiting from “… patients holding up a mirror to us” (R2:21).

2) Empower patients

A further reason for involvement arose from the empowering effect of involvement for patients. The researchers were of the opinion that patients should have a voice in research and be able to contribute their expectations: “I think […] that patient boards will be increasingly involved in research […], simply to give patients a voice as well. So that they get heard, […] so that they can say, what they expect […]. So, I think that is simply important. I find it really great.” (R5:41). Besides patients getting heard in research, an important aspect of the empowerment within the board was also “that they [patients] can inform themselves in-depth” (R5:42).

3) Enhance professional development

Researchers, who also work as physicians, hoped to enhance their professional development by learning about patients’ perspectives. “And the other [reason] is, that I hope, especially as a man, [to learn] explicitly from the involved patients, women, […] how the suffering actually is, what is their perspective when they visit the physician […] and to see how I can transfer that to my medical practice” (R4:3). In general, researchers aimed at improving their medical actions and the communication between patients and physicians.

4) Meet the formal demand for PPI

An increasing demand for PPI in research by external donors and high-listed journals was also mentioned as a motivational factor for involvement: “Time and again we notice that it [involving patients] is getting increasingly important” (R1:3). Most researchers rated the formal aspects as being relevant, but emphasized that content issues were their main motive. The formal demand was experienced as both an obligation to close the gap to more advanced countries, as well as an opportunity. Hence, being able to fulfill the demands may “raise the chance for funding of own projects” (R3:33) and proof of one’s own expertise in PPI could be a competitive advantage. Furthermore, the formal demand for involvement, “that one is urged to remember it [patient involvement]” (R2:27), could be useful to enhance the main objective, that is, to ensure high quality research.

5) Learn about PPI

Researchers aimed to learn about PPI processes and opportunities, with one of them putting it forward as the main reason for his involvement. According to him, working at the interface between science and clinical studies on the one side, and their practical application in medical action as a physician on the other side, made PPI a critical link between both his fields of activity. In his view, being involved in the patient board offered him the opportunity “to approach the topic and then perhaps gain a qualification and be a pioneer, an idea provider for that” (R3:17).

### Considerations regarding involvement

Patients and researchers considered various aspects before they decided to get involved in the patient board.

#### Patients

For patients, organizational aspects were important to consider as they had other obligations and needed to make time for the board activities. “If the scheduling would not work […], too short-notice or too much effort […] a whole day and out of town […], then I would probably not do it” (P5:7). Knowing dates early in advance and having meetings in a central location was emphasized as being important. For patients recruited via the online advertisement, the question of integrity and origin of the project was an important one before they agreed to get involved. The fact that they could contact the project team, received written information material and that it was a university project helped to allay their doubts. While none of the patients had content-related considerations against being involved in the patient board, one patient described the uncertainty she had regarding her potential participation: “I have no idea what will come up. Since I have never participated in such a board, I don’t know what would be expected of me.” (P4:29).

#### Researchers

Researchers mainly considered the personnel outlay that comes with actively involving patients. Not only do patients need to be found, attending the board meetings and working with the supplemental ideas that arise from the board meetings also takes time. As one researcher put it, this requires “additional effort […] somebody needs to do it, and it costs money and time, which is not that banal” (R1:19). The fact that at that time there was additional capacity available made it a unique opportunity to begin with the involvement. While considerations regarding the feasibility of the board were mentioned, e.g., functioning of communication and limited time frames for decisions, the benefit of PPI was never questioned. The researchers’ experience from the previous trial they had conducted, in which no PPI took place, enhanced the idea to actively involve patients. They found that “somehow something was missing” (R2:3), as the results were not able to capture the patients’ experiences exhaustively.

## Expectations

The expectations mentioned by patients and researchers regarding the work on the patient board concerned their roles, the topics and methods, as well as the communication and culture within the board (see Table 3).

**Table 3:** Expectations for involvement

	Patients	Researchers
Roles	• Saw own role in sharing perspectives and engaging in discussions • Wondered what would be expected from them and what influence they could have • Wondered about counterpart in patient board	• Own role expectations ranged from rather passive supporting force to active engagement • Hoped for collaboration on equal basis
Topics and methods	• Unsure about the direction of the patient board but willing to be surprised • Mostly not able to specify topics they want to discuss	• Described concrete topics for patient board • Patients should be available for question, feedback and discussions
Communication and culture	• Having results at the end of a meeting as important factor for satisfaction • Hoped for open, respectful exchange • Opinions shall be heard	• Wished for fixed structure to ensure regular communication • Hoped for open exchange and good feedback culture

### Roles

#### Patients

While patients saw their own roles in sharing their perspectives and engaging in discussions, they wondered what would be expected from them and what influence they could have. They imagined becoming part of a group of empathic women, who are in the same situation and are open to discuss UTI experiences. They however had many questions about their counterparts in the patient board: “Will researchers be present at all?” (P5:13), “Who are the ‘researchers’?” “Are they all prospective physicians or rather people that are active in research?” (P2:29), “[…] the patient board, will it actually talk to patients or does the patient board only talk to the patient board?” (P1:33). Further, some patients had uncertainties differentiating between being a (passive) test person in a clinical trial and being actively involved in the research process. In addition, they thought that the agenda setting and structure of the patient board would be determined by the external researcher, and that researchers would introduce the “scientific character” (P4:13) by presenting research findings, medical issues, etc.

#### Researchers

The researchers expected the “patients” to be a group of women potentially affected by UTIs, who would like to get together and contribute a lay perspective as counterparts to the researchers. The patients were partly described as representing the study population. While it was decided from the beginning that the coordination of the board would be done by the responsible external researcher, the researchers’ expectations for their own roles were diverse. Some saw their roles rather passively (e.g., someone else is to discuss the topics on the board), others wanted to “start a conversation” (R4:34) with the patients and have a “regular exchange” (R4:3). The researchers’ expectations included examples where the topics for the board are introduced by the researchers as well as where patients take the initiative to set the agenda.

Researchers reflected on hierarchies and hoped that the collaboration would be on an equal basis. One researcher reflected that patient roles on the patient board would differ from those of patients visiting a physician. On the board they are “totally independent” (R5:43) and free to share their perspectives unbiased; they are given a voice.

### Topics and methods

#### Patients

When asked for the topics they would like to discuss on the patient board, patients once again mostly mentioned their aims and interests for involvement. They wanted to share and discuss their experiences in a group, introduce the problems and interests they have as affected persons, improve UTI treatment and give feedback to physicians. Most patients were not able to specify the topics they wanted to discuss on the patient board. They were unsure about the direction the patient board would take, but were willing to be surprised: “I don’t know where this leads, what the aim of the whole project is. Therefore, I would take it as a surprise and see where we are heading” (P1:7). Noteworthy was the difference between two patients: one hoped for the patient board to be “an exciting information round, where everybody tells a bit about oneself and one […] gets tips from the other women” (P10:15) while another emphasized that “it [the patient board] is not an information event. It is about our perspectives” (P5:19). Only three patients named concrete topics: To discuss what was to be researched, to get insight into the trial. to consider its processes and to develop questionnaires.

#### Researchers

The researchers described concrete topics they would like to cover on the patient board. They wanted to:

- check the relevance of the trial’s theme, aims and outcome measures with respect to patients’ priorities,
- engage patients in the development and examination of trial documents,
- learn about patients’ perspectives regarding participation in clinical trials and the recruitment process of the UTI trial,
- use their views to supplement the analysis of outcomes,
- translate the results into daily life and disseminate them to the target population.

In total, researchers said the patients should be available for questions, feedback and discussions and contribute their perspectives, ideas and critique. While most topics are quite practical, researchers were uncertain if involvement in the analysis phase is too complicated and if “the translation of numbers into real life experiences […] would be the best task for the patient board […]” (R2:8).

Retrospectively, the researchers wished the patient board had been involved in the whole design stage of their UTI trial: “A lot of things for which the patient board could often have been asked for feedback […] were developed beforehand […] and were finished by the time the patient board was established” (R2:25). While some adaptations were still possible in the current trial, the researchers also planned to use the feedback of the patient board for the development of future projects. The researchers went on to say that in future trials PPI should start with the design stage of the trial and be a natural part of the research process. Involving patients’ perspectives does not come naturally for all of them yet, so some said that they still have to remind themselves of this opportunity and remember to share current topics with the patient board.

### Communication and culture

#### Patients

While patients rarely had concrete themes the board should cover, their expectations for the culture within the patient board were more detailed. They aimed for a productive and constructive cooperation. Having results at the end of a meeting was described as a factor for satisfaction – “that one has the feeling, that was a good meeting […] and one was able to make contributions” (P1:15). Patients hoped for the exchange to be “open and honest” (P1:15), “respectful […] and confidential” (P6:29–30) as well as “emphatic” (P10:15), so “that everyone is free to share his opinion” (P6:29) and “the opinions are being heard” (P1:15). Many emphasized how curious they were and how much they were looking forward to start the patient board.

#### Researchers

As four of the five researchers did not live in the city where the board meetings took place and the coordinator worked, researchers were not in daily contact with the patient board. To ensure regular reciprocal communication they wished for a fixed structure and drafted first ideas. These were for instance that the board could send regular mail updates on the board meetings to the researchers. Regarding the flow of information from the trial team to the patient board members, the researchers, however, had different ideas. While one researcher thought the communication would be ensured by the researcher on site – “[…] that will work anyway through X [member of the trial team]” (R1:37) –, another described it as her own responsibility to remember to communicate current processes to the patient board and to engage with them. She felt that this might be “the sensitive issue, that in daily routine, when one has to deliver things under time pressure […], that this [the communication to the board] may get lost” (R2:17).

Further, the researchers thought that a pleasant discussion atmosphere as well as mutual respect and a good feedback culture would enable an open exchange within the patient board. This includes respecting patients’ input, even if this does not align with the researchers’ priorities, as well as patients respecting the researchers’ work.

## Discussion

The aim of our study was to investigate the motivation and expectations of patients and researchers when getting involved in PPI for a clinical trial. We established a patient board for an RCT on UTIs and conducted telephone interviews with all involved patients and researchers.

Interviewing both the women with UTI experiences (‘patients’) and the members of the trial team enabled us to compare their motivation and expectations. The following recommendations derived from our results on aspects to be considered in planning or conducting PPI are aimed at all stakeholders that could initiate PPI. These comprise patients as well as researchers and their organizations.

### Motivation

#### Reasons and aims for involvement

Researchers mainly aimed at improving research, whereas patients wanted to advance the development of UTI treatments. Although the groups emphasized different aspects, they did not contradict each other. Moreover, patients and researchers were both interested in researchers, respectively physicians, getting deeper insights into patients’ perspectives so as to improve medical treatment. The motives to improve research and treatments are in line with the substantive, results-driven argumentation for conducting PPI and are also consistent with the reasons for PPI that have been anecdotally reported in other studies [4, 14, 17, 18, 34].

Patients supported the idea of being empowered by getting a voice in research. Among the researchers, only one explicitly spoke about the need for PPI to empower patients. This difference may influence the way patients and researchers understand their roles in PPI and the way decisions are made within the research process.

Whereas patients saw the patient board as an opportunity for exchange about UTIs with peers and practitioners, only one of the researchers mentioned this aspect and emphasized his intention to be available for patients’ questions. Exchange on the respective health condition hence seems to be an important motive for patients. It is however not mentioned in the general rationales for PPI and does not seem to be the main focus of researchers. Nevertheless, it may be advisable for stakeholders to reflect on how the wish for exchange can be reasonably integrated in the conduct of PPI. In our PPI we underestimated this interest and later had to fit in time slots to allow for longer exchange during the board meetings.

Some patients wanted to learn about the conduct of clinical studies and/or group discussions through their involvement in the patient board. In contrast, most researchers aimed at gaining knowledge on PPI. These motives are not part of the general argumentation for conducting PPI, but are rather aspects that can be achieved as secondary positive side effects.

Another reason given by the researchers for their involvement in the patient board was the increasing demand for PPI. This finding has also been reported in earlier studies on PPI [21, 34, 35]. In our telephone interviews researchers seemed to feel the need to justify this motive, repeatedly emphasizing that the formal demand was not their main reason for involvement. This raises two questions. Firstly, how free were the researchers to talk about their actual motivation in the telephone interviews? And, on a larger scale, if researchers feel a permanent demand for PPI and the need to justify their involvement in PPI, how does this influence their attitude towards PPI? The external demand to conduct PPI (so that the criteria for donation or publication are met) might have an influence on the genuine interest of researchers in PPI. Further, when aware of this external demand on researchers, patients may get concerned that their involvement is a mere token involvement. To avoid misconceptions, researchers should reflect early on their motives and discuss them openly with patients.

#### Considerations for involvement

Both groups named organizational aspects, such as the time to be invested, the centrality of location and a long-term planning of meetings. Furthermore, they were all interested in receiving detailed information beforehand. While researchers wanted to know about their tasks and obligations in PPI, patients wanted information about what to expect on the board (especially in contrast to participating as test persons in clinical research). For patients it was essential to get involved in a trustworthy project. Especially as PPI was completely unknown to them, the way they were contacted had an impact as they had trust in their counterparts (GPs, respectively member of local university). As the aspect of integrity was emphasized a lot by patients, stakeholders looking for people to involve in PPI should take this aspect into account.

### Expectations

#### Roles

We found that the researchers’ argumentation for PPI strongly built on its expected usefulness. They saw PPI as a means to make research better by enhancing its relevance and quality. Patients shall be available to react to researchers’ questions, and bring in their experiences and opinions. What could this possibly mean for the roles of the involved patients and researchers? According to Ives et al. [6] this approach may be “[…] similar in nature to transaction, in which the services of one party are contracted by another in order to bring necessary skills to the project”. If PPI is seen as a contracted service, this may influence the character of the cooperation, e.g. with regard to decision-making. When asked for their expectations towards the patient board, researchers did not mention patients participating in decisions. However, this does not necessarily mean that patients were not allowed to do that. The fact that patients received allowances for their involvement may also contribute towards giving the impression of a contracted service and raise questions on the economy of PPI. For some patients the allowances were an additional incentive for involvement, for others they were a mere gratification or a sign of appreciation for the time they invested. None of them described the allowances as payment for a contracted service. The allowances for the patients were not a topic researchers discussed in the telephone interviews, although they were informed about it. Viewing PPI as a service may conflict with empowerment in the sense that if patients were to be involved only to provide a service, then their empowerment would not be a priority. Their role then might be more the role of an observer than an active actor in the conduct of research. This may be even more the case in a trial such as the one we conducted, in which the design was largely decided before the involvement started.

The idea of being able to make a difference seemed to be a strong motivator for patients. However, most of our patients were not sure how realistic this aim was and what their exact roles and tasks would be. They were also unsure about whom and what to expect in the patient board. Roles and tasks should therefore be discussed at the beginning of PPI.

While working collaboratively on the trial would be a strong motivator for patients, a perception that this is not matched by an equal interest from the researchers would be a strong disincentive for patients to get involved.

Some members of the trial team saw their own role as a “supporting force” (R2:5), who does not necessarily participate in all patient board meetings but would get informed about the results. Perhaps the fact that the coordination of the patient board was with a person not involved in the trial had an impact on the way PPI was understood by researchers. This may be different in situations where one of the trial coordinators also coordinates the PPI for the trial. However, this form of conducting PPI with an independent researcher as coordinator includes the risk that the benefit of PPI suffers from (too) little interaction between patients and researchers. As Staley pointed out, the impact of involvement on research links to the impact the involvement has on the researchers: The interaction with patients allows researchers to learn and change their thinking [36]. This results in researchers doing research differently. Thus, if PPI does not allow for enough interaction between patients and researchers, it might not have that big of an impact and might have more resemblance to qualitative research, in which data is collected [37].

#### Topics and methods

Researchers said patients should be involved in the design of the trial, development of trial material, preparation of recruitment, analysis of outcomes and dissemination of results. However, for the phases during which recruitment and data collection take place in the RCT, they said the patient board was to be involved only in the event of problems. Therefore, there may be times in the conduct of the RCT during which there are no issues for discussion within the patient board. Stakeholders planning PPI should be aware that this might also occur in other studies and should think about how to fill potential ‘gaps’ in the PPI. It might be more advisable to involve patients at all stages of the research process and not only in the event of problems. Researchers cannot anticipate in advance what patients would add to the trial, they “[…] don’t know, what they don’t know” [36].

Patients had few suggestions regarding which contents to cover in the board. Although they received information on clinical research and the RCT concerned, they reported that they lacked knowledge about research processes. Some researchers expected the patients to take the initiative to set the agenda or at least to bring in their own topics. Stakeholders that initiate PPI should hence reflect on how patients can be empowered to contribute topics. This may be especially relevant when patient involvement starts after the design of the trial, as the patients are confronted with a finalized design and a research team that is already familiar with each other.

#### Communication and culture

Both groups had similar expectations regarding the culture of cooperation within the board. They hoped for a pleasant, open atmosphere and emphasized the value of mutual respect.

While patients wondered if researchers would be present at the patient board meetings, most researchers imagined that they would not always be able to get involved in-person as they had a two-hour ride to the patient board. Therefore, they found it important to reflect on ways of communication from the beginning onwards.

### Limitations of this study

We conducted semi-structured telephone interviews with all ten patients and five researchers involved in the patient board. We did not talk to patients that fulfilled the requirements but decided against getting involved, (e.g. when asked by their GP) as we did not have access to them. This limits our results to patients for whom the positive aspects outweighed the negative aspects. We further do not know how many decliners we had and why they decided against getting involved.

The researchers that took part in our patient board may have an above-average interest in PPI. They already made efforts to involve patients’ perspectives earlier in the trial and invested time and funding in the conduct of the patient board. While the anecdotal evidence from the literature indicated that the view on PPI may vary between it being a value in itself and being a burden [21–23], none of the researchers in our study described having a negative view on PPI.

Most of the researchers that got involved in the patient board were also working as physicians and had practical experiences with UTIs. It is likely that their perspectives are already closer to the perspectives of patients than the perspectives of researchers who exclusively work in research.

As the patient board was established after the trial was approved for funding, questions on trial design were already decided without including patients’ perspectives. This may, however, be a common challenge that PPI only starts after research funding is approved, as the costs for PPI (e.g. for allowances and location) during the application phase have to be provided by the researchers or their organizations. Nonetheless, it would be advisable to involve patients’ perspectives from the beginning [38]. Even when financial resources are scarce, it may still be possible to find patients to get involved, as we found that some patients would still have gotten involved without allowances. However, such an approach may lead to a less heterogeneous group of patients involved.

As we specifically looked for people interested in being on the patient board and taking part in the qualitative study – that is, highly motivated people, selection bias could have affected our study. The condition to get involved in the patient board and take part in the qualitative study may also have influenced the set of patients towards people interested in learning about research methods and group discussions. For the UTI trial, one of the inclusion criteria was an age between 18 and 75 years. Within the board, however, we did not involve patients that were over 62 years old. Therefore we do not know if older patients would have specific motives or expectations when getting involved in PPI [39].

Further, we cannot exclude that patients and researchers gave socially desirable answers, e.g. with regard to the allowances (patients) or the formal demand for PPI (researchers). The interviews with patients were short with twelve minutes mean to cover both their motivations and their expectations. Patients were free to talk as long as they wished. However, as the work in the patient board was unknown to them, it might be that it was difficult to articulate specific expectations.

We designed this study on patients’ and researchers’ motivation and expectations regarding PPI without involving patients at this stage and thereby might have missed the opportunity to involve their perspectives and concerns in the design phase. We involved patients in the analysis and the discussion of the results, and we will involve patients in the dissemination of the results. Besides co-authoring this article with patients, we aim to present the results at a conference together and to hold a workshop on PPI with researchers and research funders that will be co-hosted by patients.

## Conclusion and recommendations

In recent years it has increasingly been recognized that patients should be involved in the design, conduct and dissemination of research. Besides empowering patients and democratizing research, involvement is expected to enhance the quality of research and the development of equitable healthcare solutions. The implementation of involvement can be hampered by differing motivation and expectations among the parties involved.

Our results demonstrate that patients’ and researchers’ motivation to get involved arose from various reasons. Some, such as the aim to improve research or support the idea of patients getting a voice, were similar to the general normative and substantive rationales for PPI. Others, e.g. the exchange with peers and scientists, the monetary incentive, the professional development and the formal demand for PPI, were not covered by the general rationale for PPI, but confirmed the anecdotal knowledge on motives for PPI aggregated from previous studies.

In some regards patients’ and researchers’ motives and expectations were similar, e.g. their aims to improve research and treatments for UTIs, and their ideas of how to work in the patient board. In other regards there were differences, e.g. in their expectations for agenda setting and in the understanding of their roles. Stakeholders who conduct PPI should keep the latter in mind to enhance successful involvement. Discussing the roles and tasks of the involved patients and researchers at the beginning is necessary to develop a common understanding of PPI and to address patients’ insecurities. Stakeholders might need to reflect on how to align patients’ wishes for exchange and empowerment with researchers’ service-like expectations for PPI topics and cooperation. Enabling patients to introduce topics for discussion is necessary to ensure a balanced exchange on patient-centered issues throughout the trial’s conduct.

In each study the enabling factors for good cooperation and successful involvement will be different. We found it beneficial to get to know the motivations and expectations of all parties involved at the start. Comparing the perspectives of all parties involved can reveal potential conflicts, help avoid disappointments and hence ensure that the motivation remains.

## Additional files

Additional file 1: COREQ (COnsolidated criteria for REporting Qualitative research) checklist, PDF file. (PDF 6219 kb)

Additional file 2: GRIPP2 Checklist, PDF file. (DOCX 107 kb)

## Abbreviations

COREQ: Consolidated criteria for reporting qualitative research
GP: General practitioner
PPI: Patient and public involvement
UTI: Urinary tract infection
